# Interplay between genetic predisposition, macronutrient intake and type 2 diabetes incidence: analysis within EPIC-InterAct across eight European countries

**DOI:** 10.1007/s00125-018-4586-2

**Published:** 2018-03-17

**Authors:** Sherly X. Li, Fumiaki Imamura, Matthias B. Schulze, Jusheng Zheng, Zheng Ye, Antonio Agudo, Eva Ardanaz, Dagfinn Aune, Heiner Boeing, Miren Dorronsoro, Courtney Dow, Guy Fagherazzi, Sara Grioni, Marc J. Gunter, José María Huerta, Daniel B. Ibsen, Marianne Uhre Jakobsen, Rudolf Kaaks, Timothy J. Key, Kay-Tee Khaw, Cecilie Kyrø, Francesca Romana Mancini, Elena Molina-Portillo, Neil Murphy, Peter M. Nilsson, N. Charlotte Onland-Moret, Domenico Palli, Salvatore Panico, Alaitz Poveda, J. Ramón Quirós, Fulvio Ricceri, Ivonne Sluijs, Annemieke M. W. Spijkerman, Anne Tjonneland, Rosario Tumino, Anna Winkvist, Claudia Langenberg, Stephen J. Sharp, Elio Riboli, Robert A. Scott, Nita G. Forouhi, Nicholas J. Wareham

**Affiliations:** 1 MRC Epidemiology Unit, University of Cambridge School of Clinical Medicine, Box 285 Institute of Metabolic Science, Cambridge Biomedical Campus, Cambridge, CB2 0QQ UK; 2 Department of Molecular Epidemiology, German Institute of Human Nutrition Potsdam-Rehbruecke, Nuthetal, Germany; 3 German Center for Diabetes Research (DZD), München-Neuherberg, Germany; 4 Department of Medical Genetics, Cambridge Institute for Medical Research (CIMR), University of Cambridge, Cambridge, UK; 5 Catalan Institute of Oncology (ICO), Barcelona, Spain; 6 Navarre Public Health Institute (ISPN), Pamplona, Spain; 7 CIBER de Epidemiología y Salud Pública (CIBERESP), Madrid, Spain; 8 Department of Epidemiology and Biostatistics, School of Public Health, Imperial College London, London, UK; 9 Bjørknes University College, Oslo, Norway; 10 Department of Epidemiology, German Institute of Human Nutrition Potsdam-Rehbruecke, Nuthetal, Germany; 11 Public Health Division of Gipuzkoa, San Sebastian, Spain; 12 Instituto BIO-Donostia, Basque Government, San Sebastian, Spain; 13 CESP, Faculty of Medicine, University Paris-South, Faculty of Medicine, University Versailles-St Quentin, Inserm U1018, University Paris-Saclay, Villejuif, France; 14 Epidemiology and Prevention Unit, Fondazione IRCCS, Istituto Nazionale dei Tumori, Milan, Italy; 15 International Agency for Research on Cancer, Lyon, France; 16 Department of Epidemiology, Murcia Regional Health Council, IMIB-Arrixaca, Murcia, Spain; 17 Department of Public Health, Section for Epidemiology, Aarhus University, Aarhus, Denmark; 18 National Food Institute, Division for Diet, Disease Prevention and Toxicology, Technical University of Denmark, Kongens Lyngby, Denmark; 19 Division of Cancer Epidemiology, German Cancer Research Center (DKFZ), Heidelberg, Germany; 20 Cancer Epidemiology Unit, University of Oxford, Oxford, UK; 21 Department of Public Health and Primary Care, University of Cambridge, Cambridge, UK; 22 Danish Cancer Society, Copenhagen, Denmark; 23 Andalusian School of Public Health, Hospitales Universitarios de Granada/Universidad de Granada, Granada, Spain; 24 Department of History of Medicine, Lund University, Malmö, Sweden; 25 Julius Center for Health Sciences and Primary Care, University Medical Center Utrecht, Utrecht, the Netherlands; 26 Cancer Risk Factors and Life-Style Epidemiology Unit, Cancer Research and Prevention Institute – ISPO, Florence, Italy; 27 Dipartimento di Medicina Clinica e Chirurgia, Federico II University, Naples, Italy; 28 Department of Clinical Sciences, Clinical Research Center, Skåne University Hospital, Lund University, Malmö, Sweden; 29 Department of Nutritional Research, Umeå University, Umeå, Sweden; 30 Public Health Directorate, Asturias, Spain; 31 Department of Clinical and Biological Sciences, University of Turin, Turin, Italy; 32 Unit of Epidemiology, Regional Health Service ASL TO3, Grugliasco, (TO) Italy; 33 National Institute for Public Health and the Environment (RIVM), Bilthoven, the Netherlands; 34 Danish Cancer Society Research Center, Copenhagen, Denmark; 35 Azienda Sanitaria Provinciale di Ragusa (ASP), Ragusa, Italy; 36 Associazione Iblea per la Ricerca Epidemiologica (AIRE-ONLUS), Ragusa, Italy; 37 The Sahlgrenska Academy, Department of Internal Medicine and Clinical Nutrition, University of Gothenburg, Göteborg, Sweden; 38 School of Public Health, Imperial College London, London, UK

**Keywords:** BMI, Body mass index, Diabetes, Diet, Dietary fibre, Genetic risk score, GRS, Insulin resistance, Interaction, Macronutrient

## Abstract

**Aims/hypothesis:**

Gene–macronutrient interactions may contribute to the development of type 2 diabetes but research evidence to date is inconclusive. We aimed to increase our understanding of the aetiology of type 2 diabetes by investigating potential interactions between genes and macronutrient intake and their association with the incidence of type 2 diabetes.

**Methods:**

We investigated the influence of interactions between genetic risk scores (GRSs) for type 2 diabetes, insulin resistance and BMI and macronutrient intake on the development of type 2 diabetes in the European Prospective Investigation into Cancer and Nutrition (EPIC)-InterAct, a prospective case-cohort study across eight European countries (*N* = 21,900 with 9742 incident type 2 diabetes cases). Macronutrient intake was estimated from diets reported in questionnaires, including proportion of energy derived from total carbohydrate, protein, fat, plant and animal protein, saturated, monounsaturated and polyunsaturated fat and dietary fibre. Using multivariable-adjusted Cox regression, we estimated country-specific interaction results on the multiplicative scale, using random-effects meta-analysis. Secondary analysis used isocaloric macronutrient substitution.

**Results:**

No interactions were identified between any of the three GRSs and any macronutrient intake, with low-to-moderate heterogeneity between countries (*I*^2^ range 0–51.6%). Results were similar using isocaloric macronutrient substitution analyses and when weighted and unweighted GRSs and individual SNPs were examined.

**Conclusions/interpretation:**

Genetic susceptibility to type 2 diabetes, insulin resistance and BMI did not modify the association between macronutrient intake and incident type 2 diabetes. This suggests that macronutrient intake recommendations to prevent type 2 diabetes do not need to account for differences in genetic predisposition to these three metabolic conditions.

**Electronic supplementary material:**

The online version of this article (10.1007/s00125-018-4586-2) contains peer-reviewed but unedited supplementary material, which is available to authorised users.

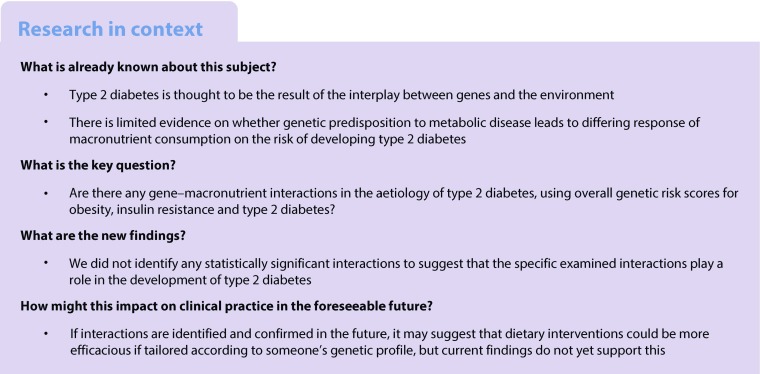



## Introduction

Genetic and environmental factors, including diet, contribute to the development of type 2 diabetes. Among dietary components, an emphasis on macronutrient composition has dominated public health dietary recommendations for decades, with guidance on the optimal per cent of energy to be consumed from carbohydrate, fat and protein. More recent dietary guidance also acknowledges the importance of macronutrient quality. For instance, evidence supporting the cardiometabolic benefits of replacing dietary saturated fat with polyunsaturated fat has led to guidance concerning fat subtype or quality. There is also a substantial genetic contribution to type 2 diabetes, with the heritability estimated to be 40–80%.

There has been increasing interest in whether this genetic susceptibility may influence how macronutrient intake affects the development of type 2 diabetes (gene–macronutrient interaction) and whether this may support the notion of ‘personalised’ or ‘precision’ nutrition. However, our recent systematic review failed to confirm any interactions via replication using similar cohorts [1]. Genetic risk scores (GRSs) may help to explain more variance for type 2 diabetes and prove better than candidate gene approaches to improve statistical power to detect potential interactions. Yet, there is a paucity of studies examining gene–macronutrient interaction using a GRS approach. Therefore, we aimed to increase our understanding of the aetiology of type 2 diabetes by investigating potential interactions between genes and macronutrient intake and their association with the incidence of type 2 diabetes using GRSs for type 2 diabetes, insulin resistance and BMI.

## Methods

### Study population and case definition and ascertainment

**Fig. 1:**
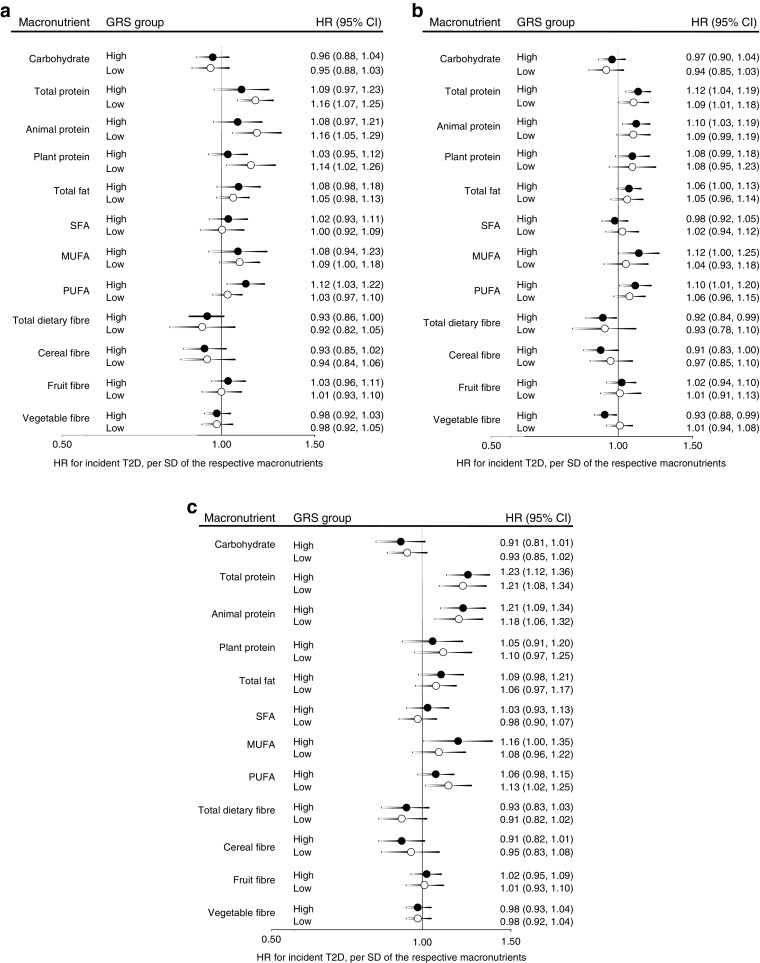
Association between macronutrient intake and the incidence of type 2 diabetes (T2D) stratified by high or low GRS for T2D (**a**), insulin resistance (**b**) and BMI (**c**): EPIC-InterAct study. GRS categorisation: T2D high ≥52, low <52 risk alleles; insulin resistance high ≥55, low <55 risk alleles; BMI high ≥91, low <91 risk alleles. Macronutrients are modelled per SD difference in intake (see Table 1 for the SD for each macronutrient). Carbohydrate intake adjusted for age (underlying time scale), sex, centre, education, physical activity, smoking status, sex-specific alcohol category, BMI, total energy intake, dietary protein, PUFA:SFA ratio, dietary fibre and first five principal components for population stratification. Intake of protein and its subtypes adjusted for age (underlying time scale), sex, centre, physical activity, smoking status, sex-specific alcohol categories, BMI, waist–hip ratio, total energy intake, dietary fibre, SFA, MUFA, PUFA, soft drinks, tea and coffee (not adjusted for carbohydrates [i.e. a substitution model]), education and first five principal components for population stratification. Intake of fat and its subtypes adjusted for age (underlying time scale), sex, centre, physical activity, smoking status, sex-specific alcohol categories, BMI, total energy intake, dietary fibre, magnesium, iron, vitamin C, leafy vegetables, tea, coffee, education and first five principal components for population stratification. Intake of dietary fibre and its subtypes adjusted for age (underlying time scale), sex, centre, physical activity, smoking status, sex-specific alcohol category, total energy intake, dietary carbohydrates, magnesium, SFA, education level and first five principal components for population stratification. Fibre subtypes were mutually adjusted. The interaction analysis for BMI GRS does not adjust for BMI. Interactions were considered statistically significant if *p* < 0.0015 (0.05/33 tests). Example of interpretation: the HR of 1 SD difference in fruit fibre on incident T2D is 1.03 in those who have the highest genetic predisposition for T2D and 1.01 for those with lower genetic predisposition for T2D. There was no statistically significant difference between those with different genetic predispositions for T2D. Black circles, high GRS; white circles, low GRS. MUFA, monounsaturated fatty acid; PUFA, polyunsaturated fatty acid; SFA, saturated fatty acid

EPIC-InterAct is a case-cohort study, nested within the European Prospective Investigation into Cancer and Nutrition (EPIC) study, described previously [2]. From 340,234 eligible participants across eight European countries, EPIC-InterAct included 27,779 healthy participants, consisting of 12,403 incident type 2 diabetes cases and a representative subcohort of 16,154 participants (including 778 individuals who developed type 2 diabetes during follow-up, according to the design of a case-cohort study). Cases of type 2 diabetes were ascertained via self-report of a diagnosis by a medical doctor or use of glucose-lowering medication noted in a lifestyle questionnaire and verified by one or more independent sources (linkage to primary and secondary care registers, medication use from prescription registers, hospital admission, mortality data and individual medical record review in some centres). The study period was from baseline (1991–1997) until the censor date of 31 December 2007. Our current analyses were based on 21,900 adults with available genome-wide genotyping and dietary data (electronic supplementary material [ESM] Fig. 1). Participants gave written informed consent and ethical approval was obtained at each participating research centre.

### Exposure and covariates

Genotyping was performed on the Illumina 660 W-Quad BeadChip (http://emea.support.illumina.com/array/array_kits/human660w-quad_dna_analysis_kit.html) or Illumina HumanCore Exome chip (http://emea.support.illumina.com/array/array_kits/humancore_exome_beadchip_kit.html) arrays, with imputation to the Haplotype Reference Consortium using IMPUTE v2.3.2 (http://mathgen.stats.ox.ac.uk/impute/impute_v2.html). All SNPs met quality control criteria for genotyping call rate (≥95%) or were well imputed (info ≥ 0.99). We generated unweighted GRSs for type 2 diabetes, insulin resistance and BMI by summing up the number of risk alleles for each trait using SNPs that reached genome-wide significance for the respective traits in published meta-analyses investigating European populations [3–5]. Habitual self-reported macronutrient intakes were estimated from country-specific baseline dietary assessments and food composition derived from the EPIC Nutrient DataBase. We examined macronutrient quantity (total carbohydrate, fat and protein intake) and quality (dietary fibre, saturated, monounsaturated and polyunsaturated fatty acids, and animal and plant protein).

### Statistical analysis

Variables with <30% missing data were imputed using multiple imputation by chained equations in Stata (v14 [StataCorp, College Station, TX, USA]) (ESM Table 1). After confirming no obvious between-imputation variation across 20 multiple imputation datasets, a single imputation was used for analyses because of computational efficiency (ESM Fig. 2). Exposures were treated as continuous variables (GRS per SD difference and macronutrient densities as 5% of total energy intake per day and 1 g/4.18 MJ [or per 1000 kcal] per day for dietary fibre) to maximise statistical power. Crude and multivariable-adjusted Prentice-weighted Cox regression models were constructed within country (for macronutrient main associations) and by genotyping chip (for GRS main associations and gene–macronutrient interactions). Given the over-representation of cases in the case-cohort analysis, the cases within and outside the subcohort were weighted differently using the weighting scheme proposed by Prentice [6]. Country-specific HRs for the variables of interest were combined across countries using random-effects meta-analysis and, where appropriate, meta-analysed across genotyping chip. Multiplicative interaction was evaluated by fitting a product term between the genetic and macronutrient exposures. For consistency, modelling was based as closely as possible on the models used in previous EPIC-InterAct analyses for carbohydrate [7], protein [8] and dietary fibre [9] (ESM Methods). Between-country heterogeneity was quantified by the *I*^2^ value and *p* for heterogeneity was derived from the Cochran-Q test.

Further secondary interaction analysis was conducted for each SNP within all three GRSs. We also examined the effect of isocaloric macronutrient substitution on these interactions using the multivariate nutrient density model (ESM Table 2).

For visualisation, we also estimated the HR for each dietary factor stratified by high and low GRS groups (Fig. 1).

Stata v14 was used for analysis. Numerical *p* values for interaction were reported; however, the threshold for determining statistical significance for interactions between GRS and macronutrient intake was ≤0.0015 (0.05/33 tests) to account for the effective number of independent tests among correlated exposures (ESM Table 3).

## Results

Table 1 shows the baseline characteristics, with more detail previously published [9], and main associations for macronutrient intake and GRSs. Positive associations with incident type 2 diabetes were observed for the proportion of energy from overall protein and animal protein intake (Table 1). However, these associations were not significant after accounting for multiple testing. No statistically significant interactions were identified–the association between the proportion of energy derived from the intake of each macronutrient and incident type 2 diabetes did not differ significantly by GRS for type 2 diabetes (*p*_interaction_ ≥ 0.20), insulin resistance (*p*_interaction_ ≥ 0.21) or BMI (*p*_interaction_ ≥ 0.22) (Fig. 1 and ESM Table 4). There was low-to-moderate heterogeneity between countries in EPIC-InterAct (*I*^2^ range 0–51.6%) (ESM Table 4).

**Table 1:** Main association between macronutrient intake or GRS and incidence of type 2 diabetes: EPIC-InterAct study

Variable	No. cases/total	Subcohort non-cases	Total incident T2D cases	HR (95% CI) per SD^a^
Median follow-up, years	9742/21,900	12.3	6.8	
Age at baseline, years		52.3 (9.3)	55.7 (7.6)	
Sex, % male		37.9	49.9	
Macronutrient intake				
Carbohydrate, % TEI	9742/21,900	44.1 (6.9)	43.7 (6.9)	0.97 (0.92, 1.02)
Protein, % TEI	9742/21,900	16.9 (3.0)	17.2 (3.0)	1.10 (1.03, 1.18)
Animal protein, % TEI	9742/21,900	10.5 (3.2)	10.9 (3.2)	1.10 (1.01, 1.18)
Plant protein, % TEI	9742/21,900	5.0 (1.3)	4.9 (1.3)	1.074 (0.999, 1.150)
Fat, % TEI	9742/21,900	34.8 (5.7)	34.7 (5.7)	1.03 (0.99, 1.08)
SFA, % TEI	9742/21,900	13.4 (3.3)	13.3 (3.3)	0.99 (0.93, 1.06)
MUFA, % TEI	9742/21,900	13.1 (3.4)	13.0 (3.4)	1.04 (0.97, 1.12)
PUFA, % TEI	9742/21,900	5.5 (1.8)	5.6 (1.8)	1.066 (0.999, 1.137)
Fibre, g	9742/21,900	22.7 (7.5)	22.6 (7.6)	0.92 (0.84, 1.02)
Cereal, g	9739/21,891	8.8 (4.9)	8.9 (4.9)	0.96 (0.86, 1.07)
Fruit, g	9608/21,611	4.3 (3.2)	4.2 (3.2)	0.86 (0.73, 1.02)
Vegetable, g	9737/21,893	4.1 (2.6)	34.0 (2.6)	0.99 (0.94, 1.04)
GRS				
T2D (per 4.3 risk alleles)	–	–	–	1.49 (1.37, 1.63)
IR (per 4.5 risk alleles)	–	–	–	1.14 (1.09, 1.20)
BMI (per 6.3 risk alleles)	–	–	–	1.07 (1.04, 1.10)^b^

Data are means (SD) unless stated otherwise

HRs for macronutrients (per SD) and incident T2D: carbohydrate intake adjusted for age (underlying time scale), sex, centre, education, physical activity, smoking status, sex-specific alcohol category, BMI, TEI, dietary protein, PUFA:SFA ratio, dietary fibre (attempt to replicate model 3 in Sluijs et al [7]); intake of protein and its subtypes adjusted for age (underlying time scale), sex, centre, physical activity, smoking status, sex-specific alcohol category, BMI, waist–hip ratio, TEI, dietary fibre, SFA, MUFA, PUFA, soft drinks, tea and coffee (not adjusted for carbohydrates; i.e. a substitution model), education (attempt to replicate model 4 in van Nielen et al [8]); intake of fat and its subtypes adjusted for age (underlying time scale), sex, centre, physical activity, smoking status, sex-specific alcohol category, BMI, TEI, dietary fibre, magnesium, iron, vitamin C, leafy vegetables, tea, coffee, education; intake of dietary fibre and its subtypes adjusted for age (underlying time scale), sex, centre, physical activity, smoking status, sex-specific alcohol category, TEI, dietary carbohydrates, magnesium, saturated fatty acids, education level. Fibre subtypes were mutually adjusted (attempt to replicate model 3 in The InterAct Consortium, 2015 [9]). HR for GRSs and T2D: adjusted for age (underlying time scale), sex, centre, first five principal components for population stratification and BMI. No. of SNPs: T2D 48 (as per Morris et al [3]), BMI 97 (as per Locke et al [4]), IR 53 (as per Lotta et al [5])

^a^SD calculated based on the whole population

^b^BMI GRS does not include adjustment for BMI

IR, insulin resistance; MUFA, monounsaturated fatty acid; PUFA, polyunsaturated fatty acid; SFA, saturated fatty acid; T2D, type 2 diabetes; TEI, total energy intake

### Secondary analysis

Results did not change substantially when: (1) using weighted GRSs; (2) modelling isocaloric macronutrient substitution (*p*_interaction_ ≥ 0.17) (see model 5 in ESM Table 2); or (3) when examining interactions with each individual SNP while accounting for isocaloric macronutrient substitution (ESM Fig. 3 and ESM Table 5). The results were similar when our current analyses based on imputed data were compared with a complete case analysis (ESM Table 6 provides an example).

## Discussion

In this large, multi-country, population-based prospective study from Europe, we found no statistically significant interactions between three metabolic GRSs and macronutrient intake on the development of type 2 diabetes. All three GRSs were positively associated with incident type 2 diabetes [3–5] and the associations between macronutrient intake and type 2 diabetes were directionally consistent with previous literature (Table 1) [7–9].

The literature on gene–macronutrient interaction studies and type 2 diabetes, using a GRS, is limited. A cross-sectional study which examined the interaction between a type 2 diabetes GRS and carbohydrate and fibre intake failed to identify interactions for prevalent type 2 diabetes (*N* = 1337 cases of type 2 diabetes) [10]. Our work is the first to examine gene–macronutrient interactions for type 2 diabetes risk prospectively using three GRSs, comprehensively investigating all major macronutrients, and consists of a large sample (*N* = 9742 cases of type 2 diabetes). The consistency across various methods (adoption of unweighted and weighted GRSs, combined GRSs as well as their constituent SNPs and application of isocaloric macronutrient substitution modelling) collectively strengthens the confidence in our null findings for interaction.

There are several factors that may contribute to the absence of interactions in our current study. Other dietary exposures, such as foods and/or dietary patterns, may offer greater insight compared with nutrients based on the food synergism hypothesis and may be subject to less accumulated measurement error. There may also be other genetic loci, with no or weak marginal genetic effects for our traits of interest, that may show a significant variation in effect between subgroups of the population. A GRS may mask interactions with individual SNPs and so may reduce statistical efficiency. Therefore, we also examined individual SNP interactions but did not identify any that were statistically significant. The generalisability of our findings is limited to European populations and research is warranted in other populations.

Among this study’s strengths, EPIC-InterAct’s prospective design minimises the potential bias due to recall bias and reverse causality for dietary exposures and the verification of diabetes cases minimises possible misclassification bias of the outcome. To our knowledge, this study represents the most comprehensive investigation of the interaction between multiple GRSs and macronutrient intake on incident type 2 diabetes, to date. We tried to address some of the key methodological issues identified from our recent systematic review, including multiple testing and inadequate control for likely confounders [1]. To reduce the risk of spurious gene–macronutrient interactions, we confirmed that the GRSs were not correlated with macronutrient intake. To our knowledge, this is also the first observational study of gene–macronutrient interactions within the cardiometabolic literature that has investigated the effect of isocaloric macronutrient substitution, which is important for public health interpretation of macronutrient density.

In conclusion, within a multi-centre European cohort, we observed no interaction between GRSs for type 2 diabetes, insulin resistance and BMI and macronutrient intake on the risk for developing type 2 diabetes. These findings suggest that currently there is no support for personalised dietary advice on macronutrient intake for type 2 diabetes prevention in subgroups of the population defined by their overall genetic risk for type 2 diabetes, insulin resistance or BMI.

## Electronic supplementary material

ESM (PDF 884 kb)

## Data Availability

Researchers seeking the analysis dataset for this work can submit a data request to the EPIC-InterAct study central contact point by emailing interact@mrc-epid.cam.ac.uk.

## Abbreviations

EPIC: European Prospective Investigation into Cancer and Nutrition
GRS: Genetic risk score
