# Supplementary material for: Interplay between genetic predisposition, macronutrient intake and type 2 diabetes incidence: analysis within EPIC-InterAct across eight European countries
Source: Diabetologia. 2018 Mar 17;61(6):1325–32. doi: 10.1007/s00125-018-4586-2 (PMC6445347; doi:10.1007/s00125-018-4586-2)
Supplement: Supplementary file 1 — (PDF 884 kb) [file 125_2018_4586_MOESM1_ESM.pdf]

Electronic Supplementary Material (ESM)

ESM Methods 1: Detailed modelling for the main and interaction analyses

ESM Table 1: Imputed baseline variables for the EPIC-InterAct Study

ESM Table 2: Multiplicative interaction between macronutrient and unweighted genetic risk scores with isocaloric macronutrient substitution: EPIC-InterAct Study

ESM Table 3: Correlations between exposures within the EPIC-InterAct Study

ESM Table 4: Multiplicative interaction between macronutrient and unweighted genetic risk scores: EPIC-InterAct Study

ESM Table 5: SNP and macronutrient interactions with pvalue for interaction<0.05

ESM Table 6: An example of interaction findings comparing multiple imputation and complete case analysis

ESM Figure 1: Flow chart of participant inclusion and exclusions, adapted from Langenberg et al., 2011

ESM Figure 2: Comparison between 20 imputed datasets for the interaction between total fibre intake and T2D genetic risk score on incident T2D

ESM Figure 3: QQ plot of pvalues for the interactions between individual SNPs for each of the genetic risk scores and macronutrients or dietary fibre on T2D (with isocaloric macronutrient substitution): EPIC-InterAct Study

ESM Methods 1: Detailed modelling for the main and interaction analyses

Statistical analyses

Variables that had <30% missing data (BMI, waist hip ratio, physical activity, education level and smoking status) were imputed using multiple imputation by chained equations in *Stata* (ESM Table 1) [1]. After confirming no obvious between-imputation variation across twenty multiple-imputation datasets [1], a single imputation was used for analyses because of computational efficiency (ESM Figure 1 provides an example). Complete-case analysis was also performed as sensitivity analysis. Potential outliers were Winsorized at the 1st and 99th percentile.

Main effect analyses

The associations between macronutrient intake and type 2 diabetes were estimated by treating macronutrient exposures as continuous variables (per SD difference in percentage of total energy intake). Crude and multivariable-adjusted Prentice-weighted Cox regression models were constructed within country. For consistency, modelling was based as closely as possible on those used in previous EPIC-InterAct analyses for carbohydrate [2], protein [3] and dietary fibre [4]. For dietary fat and subtypes (not previously published in EPIC-InterAct), we specified models adjusted for age (underlying time scale), sex, centre (nominal categorical), total energy intake (Kcal/d), physical activity (inactive, moderately inactive, moderately active, active), education (none, primary school, technical/professional, secondary school, longer education (including university)), smoking (never, former, current smoker), sex-specific alcohol intake (none, light drinking: 0.1-6g/d, moderate drinking: men 6.1-24g/d and women 6.1-12g/d, heavy drinking: men>24g/d and women>12g/d) and other dietary confounders (dietary fibre, magnesium, iron, vitamin C, green leafy vegetables, tea and

coffee in mg or g/day). Food and beverage based confounders were chosen if they may be likely confounders between macronutrient intake and type 2 diabetes but were not major sources of macronutrient intake to preserve the variance of the macronutrient which the food represented. The variable 'total energy intake' included energy from carbohydrate, fat, protein and alcohol. The covariates used can be found in the legend of Table 1. Country-specific hazard ratios (HR) for each macronutrient intake were combined across countries using random-effects meta-analysis.

For the association between GRSs and type 2 diabetes, the GRSs were treated both as continuous (per SD difference) and dichotomised exposures (as high and low GRS estimates based on being above or below the median estimates among those in the subcohort) (Table 2). Prentice-weighted Cox regression models were constructed within country and by genotyping chip. Genotype chip-specific and then country-specific estimates were combined using random-effects meta-analysis. Analyses were adjusted for age (underlying time scale), sex, centre, the first 5 principal components for population stratification and BMI.

Interaction analyses

For the interaction analyses between each macronutrient intake and each GRS on the risk of developing type 2 diabetes, both exposures were treated as continuous variables (GRS per SD difference and macronutrient as densities, being 5% of total energy intake/day and 1g/1000kcal/day for dietary fibre) to avoid loss of statistical power from categorisation. Multiplicative interaction was evaluated by fitting a product term between the GRS and macronutrient exposures [5]. Regression models were constructed in the same way as in analysis of the association between GRSs and incident type 2 diabetes, and the list of covariates included in the models were the same as for the main associations between macronutrient intake and type 2 diabetes (described above), with addition of the first 5 principal components for population stratification. Between-country heterogeneity was

quantified by the I^2 value and *P* for heterogeneity was derived from the Cochran-Q test. BMI was a covariate in the interaction analysis for IR and type 2 diabetes GRSs only.
For visualisation, we also estimated HR for each dietary factor stratified by high and low GRS groups (Figure 1).

Given that GRSs may mask interactions with individual SNPs, further secondary interaction analysis was conducted for each SNP within all 3 GRSs. We also examined if a potential effect of substitution of a macronutrient for another was modified by genetic predisposition while energy intake was held constant (i.e. isocaloric macronutrient substitution using the multivariate nutrient density model) [6]. This was performed for energy-bearing macronutrients (not dietary fibre), with the modelling strategy provided in ESM Table 4.

Stata v14 (StataCorp LP, Texas, USA) was used for analysis. Numerical p value for interaction were reported in tables and figures however, the threshold for determining statistical significance for interactions between GRS and macronutrient intake (without isocaloric macronutrient substitution) was ≤0.0015 (0.05/33 tests) to account for the effective number of independent tests among correlated exposures (see ESM Table 2 for correlations) [7]. The threshold for determining statistical significance for interactions under the substitution model was ≤0.0006 (0.05/81 tests).

ESM Table 1: Imputed baseline variables for the EPIC-InterAct Study

Characteristics	% missing	Imputed
**Number**		21900
**Age (y)**	-	
**Sex (%male)**	-	
**PA level (%)**	1.1%	Y
inactive		
moderately inactive		
moderately active		
active		
**Highest school level (%)**	1.7%	Y
none		
primary school		
technical/professional		
secondary school		
longer education (inc. university)		
**BMI (kg/m^2)**	0.7%	Y
**Waist hip ratio**	7.9%	Y
**Smoking status (%)**	1%	Y
never		
former		
current smoker		

ESM Table 2: Multiplicative interaction between macronutrient and unweighted genetic risk scores with isocaloric macronutrient substitution: EPIC-InterAct Study

Macronutrient intake (5% total energy intake)	Model (*sub=substituted)	GRS for body mass index (per 6.3 risk alleles)[%]			GRS for insulin resistance (per 4.5 risk alleles)			GRS for type 2 diabetes (per 4.3 risk alleles)		
		**Beta (95% CI)**	***P***	**I^2 (%)**	**Beta (95% CI)**	***P***	**I^2 (%)**	**Beta (95% CI)**	***P***	**I^2 (%)**
**Carbohydrate**	*model 1*	0.004(-0.024,0.032)	0.779	34.3	-0.003(-0.028,0.023)	0.847	23.2	-0.003(-0.025,0.02)	0.815	1.3
	model 2	-0.003(-0.032,0.026)	0.849	34.5	-0.009(-0.042,0.023)	0.586	44.9	-0.008(-0.031,0.015)	0.502	0.0
	model 3	-0.001(-0.033,0.032)	0.961	44.5	-0.006(-0.035,0.022)	0.665	29.0	-0.007(-0.032,0.017)	0.555	5.4
	model 4				0.004(-0.023,0.03)	0.785	0.0	0.005(-0.029,0.039)	0.774	26.5
	model 5:sub with PUFA	-0.027(-0.141,0.087)	0.642	29.2	0.012(-0.138,0.161)	0.878	39.5	0.076(-0.033,0.184)	0.172	0.0
	model 5:sub with MUFA	0.012(-0.046,0.071)	0.680	0.0	0.033(-0.07,0.137)	0.528	41.4	-0.013(-0.108,0.083)	0.797	32.5
**Total protein**	*model 1*	-0.003(-0.065,0.059)	0.923	11.0	0.05(-0.007,0.108)	0.084	0.0	-0.048(-0.112,0.015)	0.136	13.7
	model 2	0.001(-0.079,0.081)	0.980	38.1	0.047(-0.013,0.106)	0.125	0.0	-0.046(-0.115,0.023)	0.194	18.5
	model 3	-0.008(-0.071,0.056)	0.815	4.0	0.056(-0.005,0.117)	0.074	0.0	-0.041(-0.123,0.04)	0.319	36.9
	model 4				0.048(-0.022,0.117)	0.180	0.0	-0.05(-0.128,0.028)	0.213	15.1
	model 5:sub with carbohydrate	0(-0.038,0.037)	0.991	57.2	0.005(-0.022,0.032)	0.727	0.0	0.005(-0.028,0.037)	0.767	20.9
	model 5:sub with PUFA	-0.034(-0.147,0.08)	0.561	29.2	0.014(-0.138,0.166)	0.857	41.5	0.068(-0.04,0.177)	0.215	0.0
	model 5:sub with MUFA	0.012(-0.046,0.071)	0.676	0	0.024(-0.087,0.136)	0.671	49.4	-0.023(-0.111,0.064)	0.601	23.8
**Animal protein**	*model 1*	0.009(-0.059,0.076)	0.805	37.3	0.027(-0.025,0.08)	0.306	0	-0.041(-0.113,0.03)	0.258	41.0
	model 2	0.007(-0.063,0.078)	0.835	35.1	0.022(-0.033,0.077)	0.437	0	-0.036(-0.107,0.036)	0.329	35.1

	model 3	0.005(-0.059,0.07)	0.873	21.2	0.036(-0.031,0.103)	0.289	24.4	-0.032(-0.116,0.052)	0.460	49.5
	model 4				0.026(-0.038,0.091)	0.425	0	-0.048(-0.136,0.039)	0.278	38.6
	model 5:sub with carbohydrate	0(-0.037,0.037)	0.992	56.7	0.006(-0.021,0.032)	0.687	0	0.003(-0.028,0.033)	0.872	12.9
	model 5:sub with plant protein	-0.041(-0.179,0.096)	0.556	0	0.07(-0.106,0.247)	0.434	12.5	0(-0.18,0.179)	0.996	12.3
**Plant protein**	*model 1*	-0.041(-0.168,0.087)	0.533	0.0	0.059(-0.112,0.231)	0.499	32.8	0.025(-0.154,0.205)	0.780	40.0
	model 2	-0.059(-0.191,0.073)	0.384	0	0.066(-0.11,0.242)	0.463	31.5	0.01(-0.176,0.196)	0.917	40.6
	model 3	-0.06(-0.197,0.076)	0.384	0.0	0.094(-0.07,0.259)	0.260	19.3	0.01(-0.171,0.191)	0.916	34.3
	model 4				0.079(-0.084,0.243)	0.342	3.6	-0.009(-0.192,0.173)	0.921	15.6
	model 5:sub with carbohydrate	0.001(-0.037,0.038)	0.974	57.2	0.002(-0.026,0.031)	0.869	7.6	0.003(-0.028,0.034)	0.855	14.7
**Total fat**	*model 1*	0.008(-0.017,0.033)	0.552	0.0	-0.005(-0.037,0.028)	0.772	32.0	0.017(-0.009,0.044)	0.188	0.0
	model 2	0.012(-0.014,0.037)	0.375	0.0	-0.004(-0.041,0.033)	0.833	41.7	0.016(-0.011,0.043)	0.248	0.0
	model 3	0.01(-0.017,0.036)	0.471	0.0	-0.007(-0.04,0.025)	0.653	22.5	0.016(-0.012,0.045)	0.267	5.9
	model 4				-0.004(-0.048,0.041)	0.869	41.2	0.011(-0.021,0.043)	0.497	0.0
	model 5:sub with carbohydrate	0.001(-0.032,0.034)	0.955	44.3	0(-0.028,0.029)	0.986	9.3	0.004(-0.03,0.037)	0.833	23.6
	model 5:sub with total protein	-0.008(-0.074,0.059)	0.821	10.9	0.049(-0.021,0.119)	0.168	0	-0.057(-0.138,0.024)	0.167	19.7
	model 5:sub with plant protein	-0.058(-0.197,0.08)	0.407	0	0.066(-0.116,0.248)	0.475	16.1	-0.014(-0.21,0.183)	0.892	24.0
	model 5:sub with animal protein	0.004(-0.061,0.069)	0.898	21.2	0.034(-0.035,0.103)	0.335	9.1	-0.05(-0.134,0.034)	0.247	33.3
**SFA**	*model 1*	0.022(-0.03,0.073)	0.407	12.2	-0.019(-0.068,0.03)	0.444	0	0.047(-0.003,0.096)	0.066	0
	model 2	0.033(-0.018,0.085)	0.208	8.5	-0.021(-0.073,0.03)	0.412	0	0.045(-0.006,0.096)	0.085	0
	model 3	0.031(-0.019,0.08)	0.228	0	-0.027(-0.08,0.025)	0.311	0	0.046(-0.007,0.1)	0.09	2.9
	model 4				-0.015(-0.089,0.058)	0.687	22.8	0.027(-0.034,0.089)	0.384	0

	model 5:sub with carbohydrate	0.001(-0.036,0.038)	0.957	54.4	0.003(-0.024,0.03)	0.85	0	0.001(-0.031,0.033)	0.941	18.7
	model 5:sub with plant protein	-0.045(-0.183,0.094)	0.526	0	0.064(-0.114,0.243)	0.479	13.4	-0.004(-0.185,0.178)	0.966	13.7
	model 5:sub with animal protein	0.002(-0.061,0.064)	0.96	14.1	0.027(-0.039,0.092)	0.425	0	-0.052(-0.14,0.037)	0.25	38.8
	model 5:sub with total protein	-0.01(-0.072,0.052)	0.753	0	0.046(-0.025,0.116)	0.206	0	-0.054(-0.135,0.028)	0.195	19.7
	model 5:sub with PUFA	-0.031(-0.146,0.083)	0.593	29.6	0.006(-0.142,0.153)	0.939	37.9	0.075(-0.033,0.183)	0.173	0
	model 5:sub with MUFA	0.013(-0.046,0.072)	0.671	0	0.035(-0.073,0.143)	0.528	45.3	-0.008(-0.105,0.089)	0.869	33.7
**MUFA**	*model 1*	0.01(-0.046,0.066)	0.724	0	-0.001(-0.079,0.077)	0.982	35.7	0.002(-0.075,0.08)	0.95	34.0
	model 2	0.015(-0.043,0.072)	0.617	0	-0.005(-0.083,0.073)	0.902	31.7	0.001(-0.083,0.084)	0.987	38.9
	model 3	0.014(-0.044,0.073)	0.634	0	-0.003(-0.088,0.083)	0.949	38.5	-0.003(-0.092,0.087)	0.954	43.8
	model 4				0.023(-0.087,0.133)	0.685	48.5	-0.016(-0.097,0.065)	0.699	15.3
	model 5:sub with plant protein	-0.045(-0.183,0.094)	0.527	0	0.054(-0.137,0.246)	0.576	21.9	-0.01(-0.197,0.177)	0.916	17.5
	model 5:sub with animal protein	0(-0.066,0.066)	0.996	22.4	0.031(-0.036,0.098)	0.37	3.1	-0.052(-0.136,0.032)	0.226	33.2
**PUFA**	*model 1*	-0.042(-0.124,0.039)	0.309	0	-0.019(-0.129,0.092)	0.742	32.9	0.036(-0.05,0.123)	0.408	0
	model 2	-0.039(-0.141,0.064)	0.459	21.9	-0.025(-0.119,0.068)	0.598	7.97	0.044(-0.047,0.134)	0.342	0
	model 3	-0.042(-0.155,0.072)	0.471	30.5	-0.019(-0.109,0.071)	0.676	1.1	0.044(-0.048,0.136)	0.351	0
	model 4				0.01(-0.139,0.16)	0.895	40.4	0.072(-0.039,0.182)	0.204	2.2
	model 5:sub with plant protein	-0.046(-0.185,0.092)	0.51	0	0.065(-0.111,0.241)	0.467	11.5	-0.004(-0.198,0.189)	0.964	22.1
	model 5:sub with animal protein	0.002(-0.059,0.064)	0.938	12.2	0.028(-0.038,0.093)	0.41	0	-0.05(-0.135,0.036)	0.255	35.3
	model 5:sub with MUFA	0.018(-0.04,0.077)	0.544	0	0.034(-0.076,0.144)	0.545	47.5	-0.01(-0.109,0.089)	0.842	36.2

**Total dietary fibre**	*model 1*	0.005(-0.005,0.015)	0.346	0	0(-0.011,0.012)	0.95	20.9	0.001(-0.009,0.011)	0.86	0
	model 2	0.005(-0.006,0.015)	0.382	0	-0.003(-0.017,0.012)	0.723	36.5	0.001(-0.01,0.011)	0.919	0
	model 3	0.005(-0.006,0.015)	0.385	0	-0.004(-0.017,0.01)	0.602	28.6	0.002(-0.009,0.013)	0.76	0
	model 4				0(-0.015,0.016)	0.951	24.5	0.001(-0.011,0.013)	0.875	0
**Vegetable fibre**	*model 1*	-0.001(-0.026,0.024)	0.921	0	-0.008(-0.045,0.03)	0.688	44.7	0.003(-0.023,0.03)	0.81	0
	model 2	0.004(-0.026,0.033)	0.802	11.4	-0.013(-0.054,0.029)	0.553	51.1	0.002(-0.025,0.029)	0.897	0
	model 3	-0.002(-0.033,0.029)	0.903	10.6	-0.009(-0.048,0.031)	0.66	41.1	0.007(-0.021,0.035)	0.618	0
	model 4				-0.011(-0.042,0.02)	0.49	2.1	0.013(-0.019,0.044)	0.433	0
**Fruit fibre**	*model 1*	0.006(-0.012,0.024)	0.53	0	0(-0.026,0.027)	0.981	40.1	-0.003(-0.029,0.022)	0.79	33.7
	model 2	0.005(-0.016,0.027)	0.637	16.2	-0.003(-0.031,0.025)	0.83	43.1	-0.004(-0.034,0.026)	0.799	45.4
	model 3	0.004(-0.015,0.023)	0.662	0	-0.006(-0.036,0.025)	0.721	47.1	-0.002(-0.036,0.033)	0.928	54.5
	model 4				0.001(-0.042,0.045)	0.948	66.5	-0.001(-0.043,0.041)	0.965	60.2
**Cereal fibre**	*model 1*	-0.003(-0.019,0.013)	0.695	0	0.003(-0.014,0.02)	0.742	0	0.002(-0.017,0.022)	0.829	17.0
	model 2	-0.006(-0.023,0.01)	0.452	0	-0.001(-0.018,0.017)	0.925	0	0.006(-0.012,0.024)	0.52	5.5
	model 3	-0.006(-0.023,0.011)	0.472	0	-0.002(-0.021,0.016)	0.795	4.2	0.006(-0.011,0.024)	0.476	0
	model 4				-0.008(-0.029,0.014)	0.489	5.5	0.006(-0.016,0.028)	0.606	9.8

Abbreviations: SFA- saturated fatty acid, MUFA- monounsaturated fatty acid, PUFA- polyunsaturated fatty acid, GRS- genetic risk score

Macronutrients are represented by per 5% of total energy intake and dietary fibre by per g/1000kcal.

Beta-coefficient for the interaction between each of the genetic risk score with the following macronutrients on incident T2D are adjusted for the following covariates.

Macronutrient

Model 1: age (=underlying time scale), sex, centre, total energy (TEI), first 5 principal component (PC) for population stratification

Model 2: model 1 + lifestyle factors- physical activity, education, smoking, sex-specific alcohol categories

Model 3: model 2+ dietary covariates (dietary fibre, magnesium, iron, vitamin C, leafy vegetables, tea, coffee)

Model 4: model 3+ BMI

Model 5: model 4+ isocaloric macronutrient substitution

Dietary fibre

Model 1: age (=underlying time scale), sex, centre, TEI, first 5 PC for population stratification

Model 2: model 1 + lifestyle factors- physical activity, education, smoking, sex-specific alcohol categories

Model 3: model 2+ dietary covariates (carbohydrate, SFA, MUFA, PUFA intake, magnesium, iron, vitamin C, leafy vegetable, tea, coffee)
Model 4: model 3+ BMI
fibre subtypes: last model includes mutual adjustment
[&] There is no adjustment for BMI for interactions models with BMI GRS
Example of interpretation: the beta-coefficient of the interaction between total fat and BMI GRS was 0.001 for incident T2D, when fat replaced carbohydrate intake. However, this was not statistically significant because the 95% confidence interval is -0.032 and 0.034.
Isocaloric macronutrient substitution: α for significant interaction< 6.17E-4 (0.05/81 tests)

ESM Table 3: Correlations between exposures within the EPIC-InterAct Study

Exposures	T2D GRS	IR GRS	BMI GRS	fat	sfa	mufa	pufa	prot	prota	protp	cho	fb	fb_veg	fb_fruit	fb_cereal
**fat**	0.01	0.01	0.00		0.71	0.70	0.40	0.01	0.13	0.37	0.61	0.30	0.03	0.20	0.29
**sfa**	0.01	0.01	0.01			0.21	0.11	0.21	0.04	0.57	0.31	0.35	0.27	0.30	0.05
**mufa**	0.00	0.00	0.00				0.03	0.15	0.21	0.12	0.52	0.26	0.03	0.01	0.41
**pufa**	0.00	0.01	0.02					0.01	0.01	0.03	0.25	0.06	0.15	0.11	0.00
**prot**	0.00	0.00	0.03						0.04	0.04	0.32	0.17	0.37	0.18	0.15
**prota**	0.00	0.00	0.02							0.25	0.47	0.05	0.24	0.11	0.25
**protp**	0.01	0.00	0.01								0.30	0.63	0.36	0.24	0.36
**cho**	0.01	0.00	0.02									0.37	0.07	0.24	0.35
**fb**	0.01	0.00	0.01										0.53	0.52	0.43
**fb_veg**	0.01	0.01	0.01											0.32	0.13
**fb_fruit**	0.02	0.00	0.01												0.13
**fb_cereal**	0.01	0.00	0.00												

**Strength of correlation**
0
0.3
0.5
0.7
1

Spearman's Rho, without direction of correlation.
Abbreviations: sfa- saturated fatty acid, mufa- monounsaturated fatty acid, pufa- polyunsaturated fatty acid, prota- animal protein, protp- plant protein, cho- carbohydrate, fb- fibre, fb_veg: vegetable fibre, fb_fruit: fruit fibre, fb_cereal: cereal fibre.
All variables treated as continuous variables
Correlations calculated based on subcohort population only (N=12749)
Effective number of independent tests:[7] for the interaction between genetic risk scores and macronutrient interactions (without isocaloric macronutrient substitution): 10.9776 (variance of the observed eigenvalues: 1.12). This estimates the independent number of tests, accounting for any correlated macronutrients.

ESM Table 4: Multiplicative interaction between macronutrient and unweighted genetic risk scores: EPIC-InterAct Study

Macronutrient intake (5% total energy intake)	Model	GRS for body mass index (per 6.3 risk alleles)[%]			GRS for insulin resistance (per 4.5 risk alleles)			GRS for type 2 diabetes (per 4.3 risk alleles)		
		Beta (95% CI)	*P*	I^2 (%)	Beta (95% CI)	*P*	I^2 (%)	Beta (95% CI)	*P*	I^2 (%)
**Carbohydrate**	*Sluijs et al., 2013*	0.001(-0.034,0.035)	0.971	50.2	0.001(-0.03,0.031)	0.970	19.1	0(-0.028,0.027)	0.976	0.0
**Total protein**	*van Nielen et al., 2014*	-0.032(-0.1,0.036)	0.351	0.0	0.062(-0.035,0.16)	0.210	37.9	-0.06(-0.162,0.042)	0.249	43.4
**Animal protein**	*van Nielen et al., 2014*	-0.022(-0.083,0.039)	0.475	0.0	0.055(-0.048,0.158)	0.293	51.6	-0.05(-0.145,0.045)	0.302	45.5
**Plant protein**	*van Nielen et al., 2014*	-0.057(-0.206,0.091)	0.451	0.0	0.004(-0.241,0.249)	0.975	49.0	-0.022(-0.185,0.142)	0.793	0.0
**Total fat**	*model 4*	0.01(-0.017,0.036)	0.471	0.0	-0.004(-0.048,0.041)	0.869	41.2	0.011(-0.021,0.043)	0.497	0.0
**SFA**	*model 4*	0.031(-0.019,0.08)	0.228	0.0	-0.015(-0.089,0.058)	0.687	22.8	0.027(-0.034,0.089)	0.384	0.0
**MUFA**	*model 4*	0.014(-0.044,0.073)	0.634	0.0	0.023(-0.087,0.133)	0.685	48.5	-0.016(-0.097,0.065)	0.699	15.3
**PUFA**	*model 4*	-0.042(-0.155,0.072)	0.471	30.5	0.01(-0.139,0.16)	0.895	40.4	0.072(-0.039,0.182)	0.204	2.2
**Total dietary fibre (g/1000kcal)**	*Aune et al., 2015*	0.004(-0.006,0.015)	0.408	0.0	-0.004(-0.019,0.011)	0.623	44.7	-0.001(-0.011,0.01)	0.919	0.0
**Vegetable fibre (g/1000kcal)**	*Aune et al., 2015*	0(-0.029,0.028)	0.973	6.8	-0.012(-0.053,0.028)	0.548	46.4	0.007(-0.021,0.035)	0.634	0.0
**Fruit fibre (g/1000kcal)**	*Aune et al., 2015*	0.013(-0.008,0.033)	0.222	0.0	-0.007(-0.036,0.022)	0.632	40.8	-0.001(-0.029,0.026)	0.927	30.2
**Cereal fibre (g/1000kcal)**	*Aune et al., 2015*	-0.005(-0.022,0.012)	0.568	0.0	-0.002(-0.02,0.015)	0.799	0.0	0.004(-0.018,0.025)	0.735	21.5

Abbreviations: SFA- saturated fatty acid, MUFA- monounsaturated fatty acid, PUFA- polyunsaturated fatty acid, GRS- genetic risk score

Beta-coefficient for the interaction between each of the genetic risk score and respective macronutrients on incident T2D, adjusted for the following covariates (as per previously published EPIC-InterAct study, see methods).

Carbohydrate age (=underlying time scale), sex, centre, education, physical activity, BMI, smoking status, sex-specific alcohol categories, total energy intake, dietary protein, PUFA:SFA ratio, dietary fibre, first 5 principal components (PC) for population stratification

Protein and subtypes

age (=underlying time scale), total energy intake (TEI), centre, and sex, smoking, education, physical activity, sex-specific alcohol categories, dietary fibre, SFA, MUFA, PUFA, soft drinks, tea, and coffee (not adjusted for carbohydrates; i.e., a substitution model), BMI, waist hip ratio, first 5 PC for population stratification

Fat and subtypes

Model 1: age (=underlying time scale), sex, centre, TEI, first 5 PC for population stratification

Model 2: model 1 + lifestyle factors- physical activity, education, smoking, sex-specific alcohol categories

Model 3: model 2+ dietary covariates (dietary fibre, magnesium, iron, vitamin C, leafy vegetables, tea, coffee)

Model 4: model 3+ BMI

Dietary fibre and subtypes

age (=underlying time scale), sex, smoking status, physical activity, education level and sex-specific alcohol categories, total energy intake, dietary carbohydrates, magnesium, saturated fatty acids, first 5 PC for population stratification, types of fibre were mutually adjusted
Trying to replicate model 3.
[&] interactions with BMI GRS does not adjust for BMI
IR GRS and protein intake interaction does not adjust for centre because of convergence issues
Example of interpretation: the beta-coefficient of the interaction between total fat and BMI GRS was 0.010 for incident T2D.

ESM Table 5: SNP and macronutrient interactions with pvalue for interaction<0.05

GRS: genetic risk score
snp: single nucleotide polymorphism
macro: macronutrient intake
submacro: macronutrient being substituted for (this is being replaced)
beta: beta coefficient
se: standard error
lci: lower confidence interval
uci: upper confidence interval
p_int: pvalue for interaction
i_sq: I squared for heterogeneity
p_het: pvalue for heterogeneity

Threshold for p value for interaction after Bonferroni correction< 9.4E-06

Analysis:
GRS per SD
Macronutrient per 5%TEI

GRS	snp	macro	submacro	beta	se	lci	uci	p_int	i_sq	p_het
T2D	rs3802177	MUFA	PUFA	-0.19363	0.053498	-0.29848	-0.08877	0.000295	0	0.947871
T2D	rs3802177	MUFA	protein	-0.18882	0.053118	-0.29293	-0.08472	0.000378	0	0.876609
T2D	rs3802177	MUFA	SFA	-0.18842	0.053497	-0.29327	-0.08356	0.000428	0	0.7951
BMI	rs1772499	protein	fat	-0.17888	0.050957	-0.27876	-0.07901	0.000447	0	0.532623
BMI	rs1772499	protein	SFA	-0.17796	0.051264	-0.27843	-0.07748	0.000518	0	0.54333
T2D	rs3802177	MUFA	carbohydrate	-0.18391	0.053222	-0.28822	-0.0796	0.000549	0	0.652556
IR	rs6937438	PUFA	protein	-0.27893	0.083807	-0.44319	-0.11467	0.000874	0	0.46869
IR	rs6937438	PUFA	SFA	-0.27669	0.083982	-0.44129	-0.11209	0.000986	0	0.43715
IR	rs6937438	PUFA	carbohydrate	-0.27449	0.084018	-0.43916	-0.10982	0.001087	0	0.46117
BMI	rs3849570	carbohydrate	SFA	-0.0574	0.01768	-0.09205	-0.02275	0.001168	0	0.578912
BMI	rs3849570	carbohydrate	fat	-0.05674	0.017515	-0.09107	-0.02241	0.001198	0	0.695759
BMI	rs3849570	carbohydrate	protein-plant	-0.05618	0.01753	-0.09054	-0.02182	0.001353	0	0.580342
BMI	rs3849570	carbohydrate	protein	-0.05506	0.0173	-0.08897	-0.02115	0.00146	0	0.658406
BMI	rs3849570	carbohydrate	protein-animal	-0.05488	0.017361	-0.0889	-0.02085	0.001572	0	0.675645
BMI	rs1928295	protein	SFA	0.133487	0.044466	0.046336	0.220639	0.002682	0	0.876518
BMI	rs1928295	protein	fat	0.130323	0.044159	0.043773	0.216873	0.003165	0	0.793691
IR	rs2249105	fibre-fruit	fruit	0.044212	0.015813	0.01322	0.075204	0.005174	0	0.815511
T2D	rs6878122	PUFA	SFA	-0.23937	0.088628	-0.41308	-0.06567	0.006916	8.402164	0.365208
BMI	rs4256980	protein-animal	PUFA	-0.11021	0.041447	-0.19145	-0.02898	0.007835	0	0.691283
BMI	rs1772499	protein-animal	MUFA	-0.14014	0.052708	-0.24345	-0.03684	0.007841	17.57618	0.291157
BMI	rs9540493	protein-plant	protein-animal	-0.26878	0.101136	-0.467	-0.07055	0.00787	0	0.882251
T2D	rs6878122	PUFA	carbohydrate	-0.24617	0.092727	-0.42791	-0.06443	0.007936	13.21932	0.326783
BMI	rs1772499	protein-animal	SFA	-0.14223	0.053795	-0.24767	-0.0368	0.008194	20.47793	0.267142
BMI	rs1928295	protein-animal	MUFA	0.105908	0.040259	0.027001	0.184815	0.008522	0	0.651991
BMI	rs1928295	protein-animal	PUFA	0.105886	0.04028	0.026938	0.184833	0.00857	0	0.59184
IR	rs1740295	fibre-cereal	fruit	-0.09136	0.034859	-0.15968	-0.02304	0.008772	0	0.537019
BMI	rs4256980	protein-animal	fat	-0.10794	0.041355	-0.18899	-0.02688	0.009054	0	0.750602
T2D	rs6878122	PUFA	protein	-0.26542	0.102168	-0.46566	-0.06517	0.009381	24.8121	0.231158
BMI	rs1772499	protein-animal	PUFA	-0.13636	0.052769	-0.23979	-0.03294	0.009761	18.32404	0.284983
BMI	rs1928295	protein-animal	SFA	0.103671	0.040459	0.024373	0.182968	0.010396	0	0.683063
BMI	rs1772499	protein-animal	fat	-0.14301	0.056116	-0.25299	-0.03303	0.010819	26.50896	0.217128
BMI	rs3101336	carbohydrate	protein-animal	0.043364	0.017025	0.009996	0.076732	0.010861	0	0.675347
BMI	rs3101336	carbohydrate	protein	0.043137	0.016993	0.009831	0.076444	0.011134	0	0.623208
BMI	rs4256980	protein	fat	-0.11525	0.04542	-0.20427	-0.02623	0.011165	0	0.744068
BMI	rs1244663	fibre-total	fruitw	0.027329	0.010785	0.006191	0.048467	0.011275	0	0.707056
BMI	rs3101336	carbohydrate	protein-plant	0.043401	0.017153	0.009782	0.077019	0.011397	0	0.657618
BMI	rs4256980	protein-animal	MUFA	-0.10428	0.041583	-0.18578	-0.02278	0.012153	0	0.652189
BMI	rs9540493	protein-plant	SFA	-0.25501	0.10177	-0.45448	-0.05554	0.01222	0	0.77929
BMI	rs9540493	protein-plant	PUFA	-0.25442	0.101563	-0.45348	-0.05536	0.012243	0	0.707261
BMI	rs6091540	fibre-vegetable	fruit	0.065897	0.026366	0.01422	0.117575	0.012444	19.60749	0.274361
BMI	rs1928295	protein-animal	fat	0.101102	0.040475	0.021772	0.180433	0.012494	0.885112	0.4224
BMI	rs7899106	PUFA	protein	0.335853	0.134785	0.07168	0.600027	0.012711	0	0.814747
BMI	rs3101336	carbohydrate	SFA	0.043032	0.017292	0.00914	0.076924	0.012827	0	0.779073
BMI	rs7164727	fibre-total	fruitw	0.020231	0.008135	0.004287	0.036176	0.012884	0	0.899224
T2D	rs780094	MUFA	protein	-0.12225	0.04934	-0.21895	-0.02554	0.013226	0	0.435059
BMI	rs9540493	protein-plant	fat	-0.25045	0.101113	-0.44863	-0.05227	0.013252	0	0.681441
BMI	rs4256980	protein	SFA	-0.11204	0.045796	-0.2018	-0.02228	0.014426	0	0.669374

GRS	snp	macro	submacro	beta	se	lci	uci	p_int	i_sq	p_het
T2D	rs2943640	protein	SFA	0.133863	0.054792	0.026473	0.241252	0.014561	3.893046	0.399966
BMI	rs9540493	protein-plant	MUFA	-0.24835	0.101956	-0.44818	-0.04852	0.014856	0	0.821749
BMI	rs2112347	fibre-cereal	fruit	-0.03464	0.014284	-0.06264	-0.00665	0.01529	10.75583	0.346583
BMI	rs4256980	protein-animal	SFA	-0.10061	0.041671	-0.18228	-0.01894	0.015759	0	0.72759
BMI	rs3101336	carbohydrate	fat	0.041177	0.017117	0.007628	0.074726	0.016147	0	0.751394
T2D	rs163184	fibre-total	fruitw	0.022496	0.009455	0.003965	0.041027	0.017346	7.170175	0.374831
T2D	rs1242735	fibre-cereal	fruit	-0.04801	0.020385	-0.08796	-0.00805	0.018524	12.70752	0.33092
BMI	rs7715256	protein	fat	-0.10242	0.044354	-0.18935	-0.01549	0.020938	0	0.921712
BMI	rs1112666	carbohydrate	fat	0.043581	0.019038	0.006267	0.080896	0.022071	0	0.600016
IR	rs308971	protein	fat	-0.22522	0.098415	-0.41811	-0.03233	0.022112	32.02021	0.172349
BMI	rs1288545	MUFA	protein	-0.09952	0.043586	-0.18495	-0.01409	0.022411	0	0.629832
BMI	rs1167827	fibre-vegetable	fruit	-0.05962	0.026133	-0.11084	-0.0084	0.022529	30.92792	0.181087
BMI	rs7899106	PUFA	carbohydrate	0.309944	0.136247	0.042904	0.576984	0.022914	0	0.780279
BMI	rs7715256	protein	SFA	-0.09984	0.044564	-0.18718	-0.01249	0.025071	0	0.756689
T2D	rs2943640	protein	fat	0.131845	0.059146	0.01592	0.24777	0.025805	15.23117	0.310415
BMI	rs492400	fibre-total	fruitw	-0.01769	0.007942	-0.03326	-0.00212	0.025922	0	0.638099
IR	rs308971	protein	SFA	-0.22108	0.099312	-0.41573	-0.02643	0.026006	31.91649	0.173175
BMI	rs1013228	fibre-fruit	fruit	-0.03489	0.015688	-0.06563	-0.00414	0.026167	6.17415	0.382543
BMI	rs9540493	carbohydrate	protein-animal	-0.04005	0.018021	-0.07537	-0.00473	0.02627	8.879879	0.361452
IR	rs731839	protein-plant	protein-animal	-0.26846	0.121924	-0.50743	-0.02949	0.027675	0	0.836634
IR	rs1113032	protein-plant	protein-animal	0.4372	0.199237	0.046702	0.827699	0.028209	27.65237	0.20772
BMI	rs1105740	MUFA	PUFA	-0.17978	0.082236	-0.34096	-0.0186	0.028806	0	0.701269
BMI	rs2365389	PUFA	protein	0.1411	0.064555	0.014575	0.267625	0.028835	0	0.806102
BMI	rs9540493	carbohydrate	protein	-0.04108	0.018858	-0.07805	-0.00412	0.029365	15.15177	0.311064
IR	rs7227237	PUFA	protein	0.192766	0.08851	0.019289	0.366242	0.029414	0	0.69817
IR	rs1113032	protein-plant	MUFA	0.400748	0.18492	0.038312	0.763184	0.030224	18.9776	0.279578
T2D	rs1020317	fibre-fruit	fruit	0.095503	0.044096	0.009076	0.18193	0.030327	56.63609	0.023846
BMI	rs1516725	MUFA	protein	-0.13684	0.063285	-0.26087	-0.0128	0.0306	0	0.724417
IR	rs2943645	protein	SFA	0.126671	0.058728	0.011565	0.241776	0.031014	14.1534	0.319204
IR	rs1113032	protein-plant	PUFA	0.430083	0.199406	0.039256	0.820911	0.031019	27.58538	0.20827
IR	rs308971	protein-animal	fat	-0.21541	0.099884	-0.41118	-0.01964	0.031038	43.96099	0.085516
BMI	rs2112347	protein-plant	protein-animal	-0.21606	0.100247	-0.41254	-0.01958	0.031138	0	0.778034
BMI	rs1105740	MUFA	SFA	-0.17822	0.08275	-0.3404	-0.01603	0.031267	0	0.697024
BMI	rs7899106	PUFA	SFA	0.296614	0.138032	0.026076	0.567151	0.031644	0	0.734206
BMI	rs1112666	carbohydrate	protein-plant	0.040774	0.019012	0.003511	0.078036	0.031981	0	0.70178
BMI	rs3736485	carbohydrate	protein-animal	-0.03689	0.017244	-0.07069	-0.00309	0.032412	3.068796	0.406176
T2D	rs459193	PUFA	protein	-0.17309	0.080923	-0.33169	-0.01448	0.032441	0	0.659472
IR	rs459193	PUFA	protein	-0.17309	0.080923	-0.33169	-0.01448	0.032441	0	0.659472
BMI	rs758747	protein-animal	MUFA	-0.09698	0.045391	-0.18595	-0.00802	0.03263	0	0.453197
IR	rs7227237	PUFA	SFA	0.189334	0.088745	0.015397	0.363271	0.032887	0	0.715738
BMI	rs2112347	fibre-vegetable	fruit	-0.04536	0.021265	-0.08704	-0.00368	0.032929	4.547563	0.395002
BMI	rs1112666	carbohydrate	SFA	0.040891	0.019214	0.003232	0.07855	0.033321	0	0.684636
IR	rs9492443	fibre-vegetable	fruit	0.054505	0.025612	0.004306	0.104703	0.033329	0	0.878501
BMI	rs2365389	PUFA	carbohydrate	0.138005	0.064865	0.010873	0.265138	0.033372	0	0.858583
BMI	rs1167827	fibre-fruit	fruit	-0.0293	0.013781	-0.05631	-0.00229	0.03349	0	0.589437
IR	rs1113032	protein-plant	SFA	0.40708	0.191568	0.031613	0.782547	0.033588	22.97541	0.246399
BMI	rs2033529	fibre-total	fruitw	0.017798	0.008389	0.001355	0.03424	0.033882	0	0.85123
BMI	rs7239883	protein	SFA	0.096176	0.045448	0.007099	0.185252	0.03433	0	0.794842
BMI	rs1288545	MUFA	PUFA	-0.09324	0.044095	-0.17967	-0.00682	0.034463	0	0.531842
BMI	rs7164727	fibre-cereal	fruit	0.030212	0.014294	0.002196	0.058228	0.03455	6.472793	0.380237

GRS	snp	macro	submacro	beta	se	lci	uci	p_int	i_sq	p_het
BMI	rs758747	protein-animal	fat	-0.09534	0.045122	-0.18377	-0.0069	0.034616	0	0.506501
BMI	rs2033732	protein-plant	fat	-0.25248	0.119701	-0.48709	-0.01787	0.034923	0	0.494788
IR	rs308971	protein-animal	MUFA	-0.21595	0.102469	-0.41679	-0.01511	0.035078	45.99866	0.073024
T2D	rs2943640	protein-plant	PUFA	0.250925	0.119207	0.017284	0.484567	0.035296	0	0.53354
BMI	rs1112666	carbohydrate	protein	0.039527	0.018814	0.002652	0.076403	0.035649	0	0.578254
BMI	rs1310732	protein-animal	PUFA	-0.16831	0.080138	-0.32538	-0.01124	0.03571	0	0.611583
BMI	rs2112347	protein-plant	MUFA	-0.21187	0.100884	-0.4096	-0.01414	0.035719	0	0.67641
IR	rs7227237	PUFA	carbohydrate	0.185651	0.088478	0.012238	0.359064	0.03588	0	0.715359
T2D	rs780094	MUFA	PUFA	-0.11104	0.052967	-0.21485	-0.00722	0.036053	5.328815	0.389039
IR	rs731839	protein-plant	SFA	-0.25653	0.122447	-0.49653	-0.01654	0.036166	0	0.832788
BMI	rs1112666	carbohydrate	protein-animal	0.039438	0.018837	0.002519	0.076358	0.03629	0	0.601009
T2D	rs2943640	protein-plant	protein-animal	0.248348	0.118649	0.015801	0.480895	0.036337	0	0.6108
BMI	rs1105740	MUFA	protein	-0.17014	0.081454	-0.32978	-0.01049	0.036731	0	0.719033
T2D	rs780094	MUFA	SFA	-0.11057	0.053025	-0.2145	-0.00664	0.037049	5.075031	0.39098
T2D	rs2943640	protein-plant	fat	0.24781	0.118944	0.014683	0.480937	0.037214	0	0.571937
T2D	rs3802177	protein-plant	PUFA	0.26333	0.126557	0.015282	0.511377	0.03746	0	0.967886
BMI	rs2365389	PUFA	SFA	0.134834	0.064822	0.007785	0.261882	0.03752	0	0.813916
T2D	rs1163439	PUFA	protein	-0.16449	0.079118	-0.31956	-0.00942	0.037615	0	0.86707
BMI	rs7239883	protein	fat	0.093767	0.045171	0.005233	0.182302	0.037911	0	0.778832
BMI	rs6091540	fibre-total	fruitw	0.017954	0.008652	0.000996	0.034912	0.037983	0	0.590972
IR	rs308971	protein-animal	SFA	-0.2035	0.098075	-0.39572	-0.01127	0.037997	40.99521	0.10514
BMI	rs1073368	carbohydrate	protein	0.038864	0.018745	0.002125	0.075603	0.038143	15.98271	0.304262
IR	rs1113032	protein-plant	fat	0.405724	0.19581	0.021944	0.789505	0.038262	26.20314	0.219652
IR	rs3864041	fibre-cereal	fruit	0.043878	0.021186	0.002355	0.085401	0.038347	37.56163	0.129674
BMI	rs2033732	protein-plant	MUFA	-0.24923	0.120373	-0.48515	-0.0133	0.038411	0	0.63274
T2D	rs2075423	fibre-cereal	fruit	0.034765	0.016815	0.001809	0.067721	0.038681	8.40805	0.365162
BMI	rs1516725	MUFA	PUFA	-0.13234	0.064093	-0.25796	-0.00672	0.038935	0	0.873454
BMI	rs1105740	MUFA	carbohydrate	-0.16969	0.082271	-0.33094	-0.00844	0.039157	0	0.721312
BMI	rs2112347	protein-plant	PUFA	-0.20801	0.101047	-0.40606	-0.00997	0.039534	0	0.710705
BMI	rs543874	fibre-vegetable	fruit	-0.05258	0.025547	-0.10265	-0.00251	0.039582	0.921854	0.42213
BMI	rs1288545	MUFA	carbohydrate	-0.09055	0.044049	-0.17688	-0.00421	0.039818	0	0.586056
T2D	rs1027833	fibre-vegetable	fruit	-0.05484	0.026719	-0.10721	-0.00247	0.040114	19.09052	0.278643
BMI	rs1073368	carbohydrate	protein-animal	0.0384	0.018741	0.001668	0.075132	0.040465	15.64165	0.307057
BMI	rs758747	protein-animal	PUFA	-0.09271	0.045254	-0.18141	-0.00401	0.040499	0	0.507591
BMI	rs1073368	carbohydrate	SFA	0.036358	0.017754	0.001561	0.071154	0.040569	6.243598	0.382007
BMI	rs758747	protein-animal	SFA	-0.09284	0.045399	-0.18182	-0.00386	0.040849	0	0.506121
T2D	rs2943640	protein-plant	MUFA	0.243358	0.119036	0.010053	0.476664	0.040912	0	0.601512
BMI	rs1516725	MUFA	carbohydrate	-0.13005	0.063632	-0.25477	-0.00533	0.040977	0	0.856815
BMI	rs2112347	protein-plant	SFA	-0.20645	0.101043	-0.40449	-0.00841	0.04103	0	0.75454
BMI	rs758747	protein	fat	-0.10038	0.049154	-0.19672	-0.00404	0.041132	0	0.747126
IR	rs308971	protein-animal	PUFA	-0.20278	0.099345	-0.39749	-0.00807	0.041233	42.77734	0.093155
IR	rs731839	protein-plant	PUFA	-0.24943	0.122234	-0.489	-0.00985	0.041294	0	0.757378
T2D	rs3802177	carbohydrate	protein-animal	0.046776	0.022928	0.001838	0.091713	0.041336	7.863979	0.369423
BMI	rs1685148	carbohydrate	protein-animal	-0.073	0.035839	-0.14324	-0.00275	0.041668	0	0.669175
T2D	rs780094	MUFA	carbohydrate	-0.11164	0.054815	-0.21908	-0.00421	0.041681	8.556442	0.363996
BMI	rs9540493	carbohydrate	protein-plant	-0.03778	0.018549	-0.07413	-0.00142	0.041695	11.75523	0.338585
BMI	rs2365389	protein-plant	PUFA	-0.28068	0.13785	-0.55086	-0.0105	0.041737	39.24422	0.117432
BMI	rs1307896	PUFA	protein	0.156906	0.077104	0.005785	0.308028	0.041852	0	0.564212
IR	rs7973683	carbohydrate	fat	-0.03961	0.019493	-0.07782	-0.0014	0.042157	0	0.693951
BMI	rs9540493	carbohydrate	SFA	-0.03608	0.017783	-0.07093	-0.00123	0.042464	5.137115	0.390506

GRS	snp	macro	submacro	beta	se	lci	uci	p_int	i_sq	p_het
T2D	rs1163439	PUFA	carbohydrate	-0.16119	0.079544	-0.31709	-0.00529	0.042717	0	0.849307
T2D	rs2943640	protein-plant	SFA	0.241325	0.11911	0.007874	0.474776	0.042757	0	0.575101
IR	rs6066149	protein-plant	protein-animal	-0.27384	0.135198	-0.53882	-0.00886	0.042817	0	0.532456
BMI	rs1516725	MUFA	SFA	-0.12951	0.063961	-0.25487	-0.00415	0.042881	0	0.848288
BMI	rs2365389	protein-plant	fat	-0.26264	0.129749	-0.51694	-0.00834	0.042947	33.4958	0.16069
T2D	rs780094	PUFA	SFA	0.204118	0.101035	0.006093	0.402144	0.043356	31.39684	0.177326
BMI	rs1310732	protein-animal	fat	-0.16173	0.0801	-0.31872	-0.00473	0.043481	0	0.69406
BMI	rs2365389	protein-plant	MUFA	-0.27825	0.137953	-0.54864	-0.00787	0.043694	39.79366	0.113523
IR	rs1113032	fibre-total	fruitw	0.034536	0.017163	0.000897	0.068174	0.044195	38.06322	0.125984
BMI	rs1288545	MUFA	SFA	-0.08908	0.044268	-0.17584	-0.00231	0.0442	0	0.534276
IR	rs1045241	protein-animal	SFA	0.105375	0.052423	0.002629	0.208122	0.044419	0	0.633459
BMI	rs1310732	protein-animal	MUFA	-0.161	0.080136	-0.31806	-0.00393	0.044535	0	0.673966
BMI	rs2033732	protein-plant	protein-animal	-0.24124	0.120086	-0.4766	-0.00587	0.044552	0	0.590287
IR	rs3864041	protein-plant	SFA	0.241496	0.120319	0.005675	0.477316	0.044735	0	0.87534
BMI	rs2365389	protein-plant	SFA	-0.27971	0.139521	-0.55317	-0.00625	0.044987	40.4119	0.109181
IR	rs731839	protein-plant	fat	-0.24417	0.122008	-0.48331	-0.00504	0.045361	0	0.769939
BMI	rs1307896	PUFA	SFA	0.154909	0.07744	0.00313	0.306689	0.04546	0	0.726588
BMI	rs1073368	carbohydrate	protein-plant	0.036103	0.018052	0.000722	0.071484	0.045505	9.664813	0.355254
IR	rs3864041	protein-plant	PUFA	0.239687	0.12008	0.004334	0.47504	0.045928	0	0.79263
BMI	rs2820292	PUFA	carbohydrate	0.133467	0.066914	0.002317	0.264616	0.046088	0	0.750275
T2D	rs849135	protein	fat	0.101289	0.050875	0.001576	0.201002	0.046488	0	0.669926
T2D	rs1163439	PUFA	SFA	-0.15774	0.079395	-0.31335	-0.00213	0.046948	0	0.819668
T2D	rs3802177	protein-plant	MUFA	0.25008	0.126256	0.002622	0.497538	0.047622	0	0.974301
BMI	rs2820292	PUFA	SFA	0.132521	0.066968	0.001267	0.263776	0.047829	0	0.80307
BMI	rs1685148	carbohydrate	protein	-0.07065	0.035739	-0.1407	-0.0006	0.048062	0	0.655832
BMI	rs2033732	protein-plant	SFA	-0.23847	0.120729	-0.4751	-0.00185	0.048239	0	0.473563
T2D	rs1125765	PUFA	SFA	0.251306	0.127262	0.001877	0.500735	0.048301	37.66341	0.128923
BMI	rs3736485	carbohydrate	protein	-0.03448	0.01749	-0.06876	-0.0002	0.048648	5.429392	0.388268
BMI	rs1685148	carbohydrate	fat	-0.07107	0.036059	-0.14175	-0.0004	0.048722	0	0.808037
IR	rs7973683	carbohydrate	protein-animal	-0.03852	0.019563	-0.07687	-0.00018	0.048939	0	0.662636
BMI	rs977747	protein-animal	SFA	-0.09591	0.048719	-0.1914	-0.00042	0.049	22.84033	0.247521
IR	rs1045241	protein-animal	MUFA	0.103252	0.052459	0.000434	0.206071	0.049041	0	0.686748
BMI	rs1172767	protein-plant	fat	0.37915	0.192756	0.001354	0.756945	0.049184	0	0.71259
BMI	rs758747	protein	SFA	-0.09722	0.049543	-0.19432	-0.00012	0.049725	0	0.707793
T2D	rs1125765	PUFA	carbohydrate	0.25551	0.130308	0.000112	0.510909	0.0499	39.75135	0.113823

ESM Table 6: An example of interaction findings comparing multiple imputation and complete case analysis

Unweighted T2D GRS (per SD) x Macronutrient (g/1000kcal)	Multiple imputation analysis results (9742 cases, 12158 noncases)		Complete case analysis results (9403 cases, 11745 noncases)	
	**Beta (se)**	***P***	**Beta (se)**	***P***
Total dietary fibre	0.001(0.006)	0.875	0.001(0.007)	0.839
Cereal fibre	0.006(0.011)	0.606	0.002(0.016)	0.902

Abbreviation: SSB: sugar sweetened beverage, BMI: body mass index, T2D: type 2 diabetes, GRS: genetic risk score, p: p value for interaction

Modelling the same as that in ESM Table 4, model 4.

Conclusion: no substantial difference in results between analysis approaches

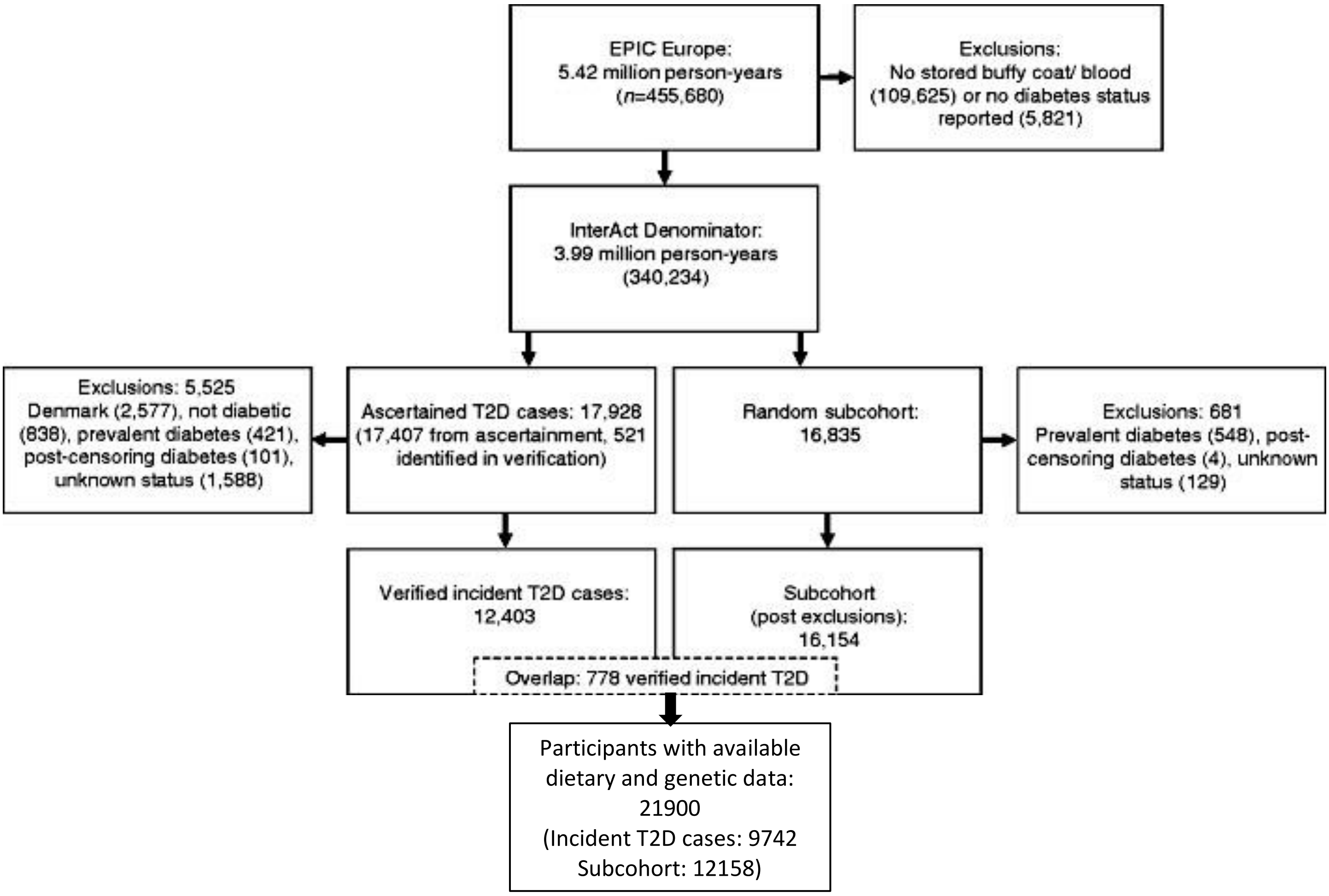

ESM Figure 1: Flow chart of participant inclusion and exclusions, adapted from Langenberg et al., 2011[8]

Reprinted by permission from Springer Nature: Diabetologia Design and cohort description of the InterAct Project: an examination of the interaction of genetic and lifestyle factors on the incidence of type 2 diabetes in the EPIC study, Langenberg et al., Copyright 2011.

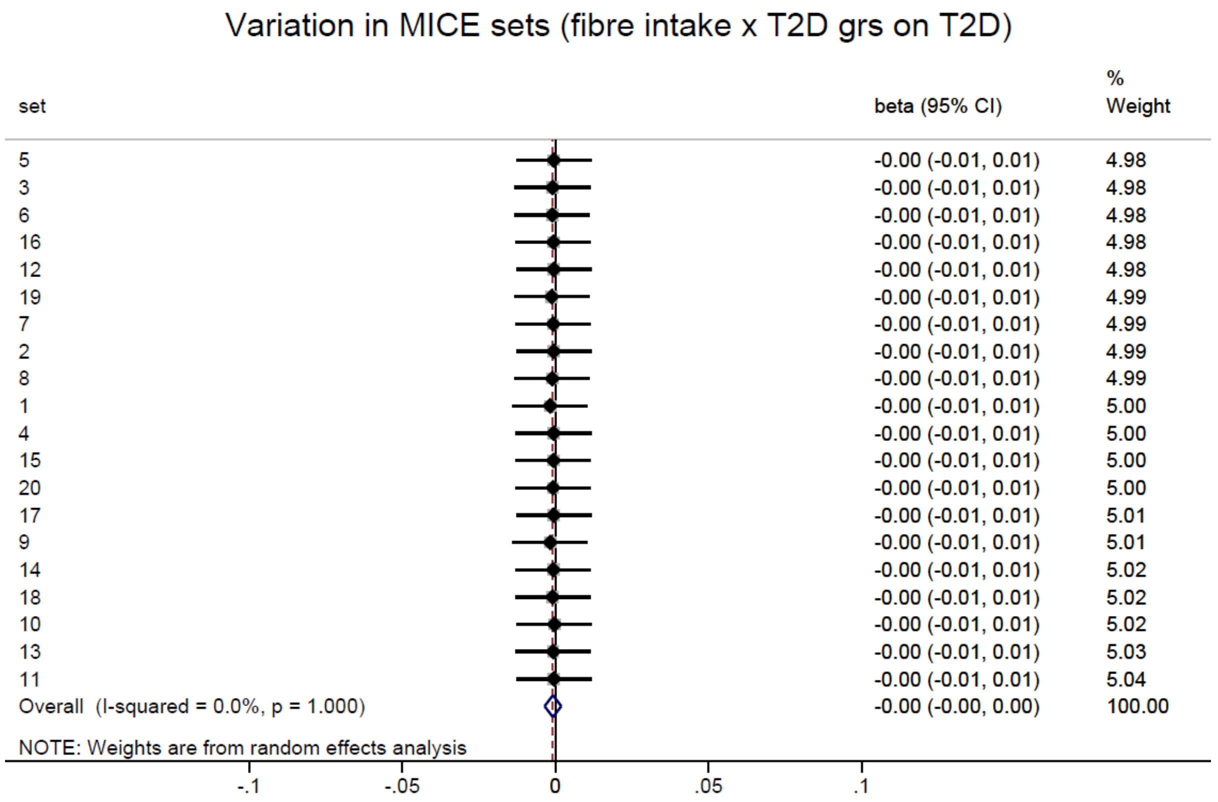

Beta coefficient of the interaction between fibre and T2D GRS on incident T2D

ESM Figure 2: Comparison between 20 imputed datasets for the interaction between total fibre intake and T2D genetic risk score on incident T2D

Abbreviation: MICE: multiple imputation using chained equations, T2D: type 2 diabetes, GRS: genetic risk score

Modelling same as model 4 of ESM Table 4. Here, analysis performed by country and pooled using random effects meta-analysis (no chip-specific analysis was undertaken).

Conclusion: total variation= within + between dataset variation is very small. Dataset number 15 was therefore chosen, at random, for all analyses presented in this paper.

within dataset variance	3.89E-05
between dataset variance	1.32E-07

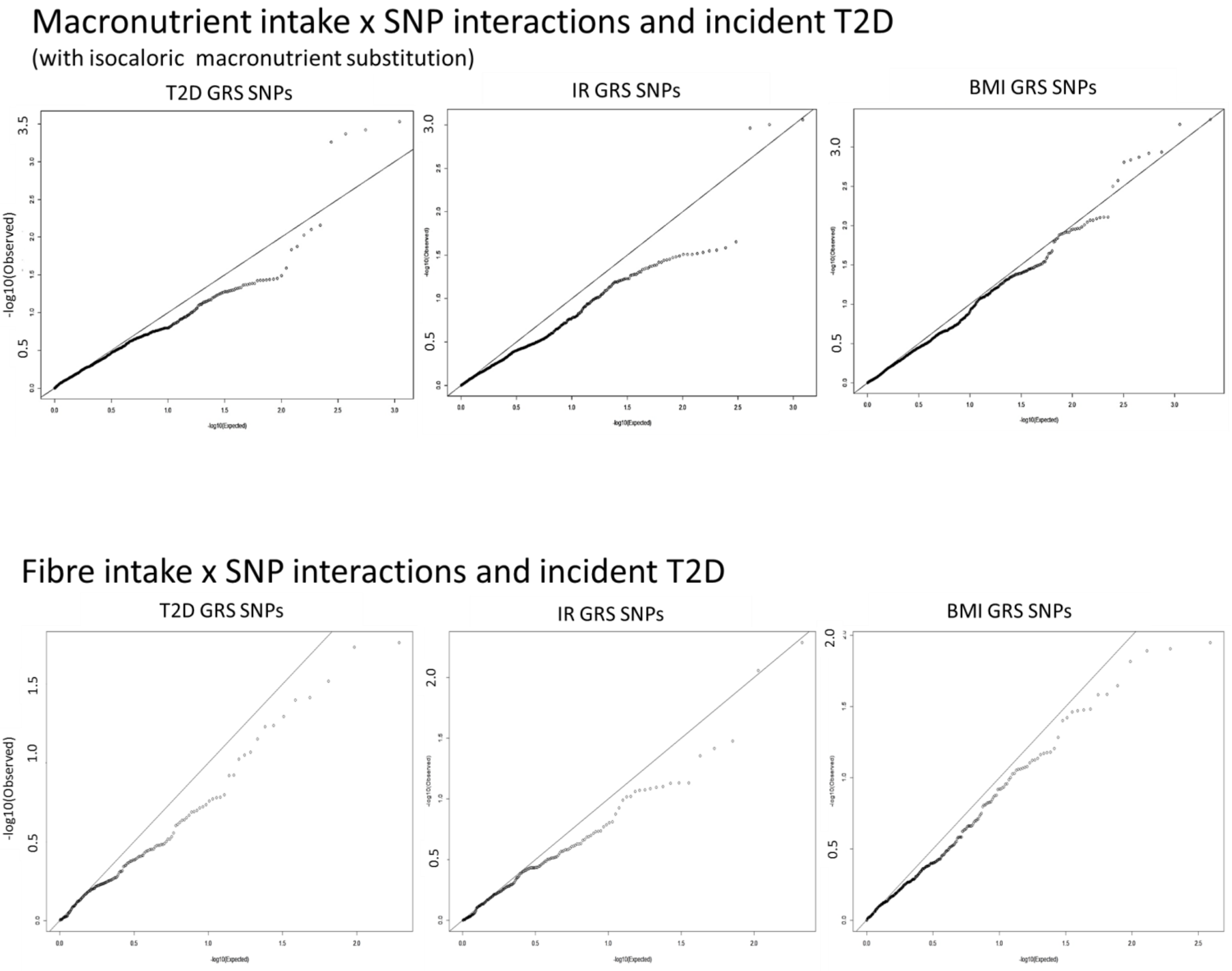

ESM Figure 3: QQ plot of pvalues for the interactions between individual SNPs for each of the genetic risk scores and macronutrients or dietary fibre on T2D (with isocaloric macronutrient substitution): EPIC-InterAct Study

All interactions are based on the most adjusted models (previously reported for ESM tables 4). None of the individual SNP interactions were significant after accounting for multiple testing. P value for interaction threshold for significance <9.50E-6 (0.05/5265 tests).

Please note that each individual GRS graph contains multiple sets of interaction analyses, therefore the expected p values may be conservative due to correlated analyses. That is, for the interaction between fibre intake and T2D GRS, this includes interactions between T2D GRS and total fibre, T2D GRS and cereal fibre, T2D GRS and vegetable fibre and T2D GRS and fruit fibre on incident T2D

A list of SNP x macronutrient interactions with pvalue for interaction<0.05 are available in ESM Table 6 (Excel)

References

1. White IR, Royston P, Wood AM (2011) Multiple imputation using chained equations: Issues and guidance for practice. Stat Med 30:377–399 . doi: 10.1002/sim.4067
2. Sluijs I, Beulens JWJ, Schouw YT Van Der, et al (2013) Dietary Glycemic Index , Glycemic Load, and Digestible Carbohydrate Intake Are Not Associated with Risk of Type 2 Diabetes in Eight European Countries. J Nutr 143:93–99 . doi: 10.3945/jn.112.165605.93
3. van Nielen M, Feskens EJM, Mensink M, et al (2014) Dietary protein intake and incidence of type 2 diabetes in Europe: the EPIC-InterAct Case-Cohort Study. Diabetes Care 37:1854–62 . doi: 10.2337/dc13-2627
4. The InterAct Consortium (2015) Dietary fibre and incidence of type 2 diabetes in eight European countries: the EPIC-InterAct Study and a meta-analysis of prospective studies. Diabetologia 58:1394–408 . doi: 10.1007/s00125-015-3585-9
5. VanderWeele TJ (2015) Explanation in Causal Inference: Methods for Mediation and Interaction. Oxford University Press, USA
6. Willett WC (2013) Nutritional epidemiology, 3rd ed. Oxford University Press, New York
7. Li J, Ji L (2005) Adjusting multiple testing in multilocus analyses using the eigenvalues of a correlation matrix. Heredity (Edinb) 95:221–227 . doi: 10.1038/sj.hdy.6800717
8. Langenberg C, Sharp S, Forouhi NG, et al (2011) Design and cohort description of the InterAct Project: an examination of the interaction of genetic and lifestyle factors on the incidence of type 2 diabetes in the EPIC Study. Diabetologia 54:2272–82 . doi: 10.1007/s00125-011-2182-9
